# Association of self-reported use of cannabis for the purpose of improving physical, mental, and sleep health with problematic cannabis use risk

**DOI:** 10.1186/s12889-023-16324-0

**Published:** 2023-08-16

**Authors:** Wura Jacobs, Ashley L. Merianos, Patrick Quinn, Jessica Barrington-Trimis, Adam Leventhal

**Affiliations:** 1 School of Public Health, Indiana University, 1025 E. 7th St., SPH 116, Bloomington, IN 47405 USA; 2 School of Human Services, University of Cincinnati, Cincinnati, USA; 3 Preventive Medicine, Keck School of Medicine, University of Southern California, California, USA

**Keywords:** Cannabis, Young adults, Motive, Cannabis use disorder, Probmelatic use, Sleep, Mental health, Physical health

## Abstract

**Background:**

Little is known about health-focused cannabis use purposes and their associations with risk for problematic cannabis use. This cross-sectional study examined three broad cannabis use purposes and association with risk for problematic use among young adult cannabis users who report using for > 1 health reasons.

**Methods:**

Young adults completed an electronic survey as part of an ongoing study on substance use and health. Those who self-reported past 6-month use of ≥ 1 cannabis products—smoking, vaping, dabbing, eating, and blunts—were included in the analysis. Their purposes for use were coded into three categories: sleep, mental, and physical health. Problematic cannabis use (PCU) was measured with the three-level structure Cannabis Abuse Screening Test (CAST-3). Adjusted multivariable regression models were used to estimate use purposes associated with with problematic cannabis use at the p < 0.05 level.

**Results:**

Participants (n = 954) were mostly female (63.94%) and Hispanic (54.93%). Mental health was the most endorsed reason (73.38%) for use among study sample. Among participants, 36.3% were classified as being at severe risk (CAST-3 score ≥ 8). There was a significant association between PCU risk and reporting cannabis use for physical health (p < 0.01), mental health, and sleep health (p < 0.01) purposes. Those who used cannabis for physical heath purposes had about four times the risk (adjusted relative risk ratio (aRRR) = 4.38, 95% CI = 3.06–6.69), those who used for mental health had about three times the risk (aRRR = 2.81, 95% CI = 1.86–4.72), and those who used for sleep health had almost two times the risk (aRRR = 1.83, 95% CI = 1.17–2.63) for severe PCU.

**Conclusion:**

All cannabis use purposes examined increased risk of problematic cannabis use. Physical health use purposes was associated with highest PCU risk. This study demonstrates the risk for cannabis use disorder associated with self-medicating with cannabis.

**Supplementary Information:**

The online version contains supplementary material available at 10.1186/s12889-023-16324-0.

## Background

In 2021, marijuana use among young adults reached historic highs since trends were first monitored in 1988 [1]. According to Monitoring the Future panel study [2], among young adults, past-year use in 2021 reached 43% while past-month used reached 29% compared to 34% and 21% respectively in 2016 [1, 2]. With recreational marijuana becoming legal in several states and jurisdictions allowing cannabis for medical purposes [3], cannabis products are increasingly varied and accessible. In recent years, the cannabis products landscape has evolved [4] to include combustibles (e.g., joints, pipes, bongs), blunts (a form of use where cannabis is rolled in a cigar leaf), vaporizers (e.g., vaping devices with THC [Tetrahydrocannabinoid] oil or wax), and edibles (food products made or infused with cannabis)[5]. Research evidence suggests this increasing availability, commercialization of products with varying tetrahydrocannabinol levels [6], and societal approval has normalized use [7] and perpetuated the idea that cannabis use is harmless [6–9].

Some of the most frequently reported health-related motives for cannabis use among young adults include mental health symptoms such as anxiety, depression, and other psychiatric symptoms [10], help with sleep disturbances, and pain alleviation [11]. However, there is unclear evidence on the safety and efficacy of cannabis use as a therapeutic for these purposes [12]. Although cannabis and cannabinoids have been found to be potentially beneficial for treating nausea and vomiting during chemotherapy, childhood epilepsy and chronic pain [3, 13], there is also evidence showing harms from acute intoxication. Chronic use is also linked to impaired driving and learning capacity, poor educational and social outcomes, and adverse fetal outcomes [14, 15]. Vaping cannabis reduces exposure to smoke-related toxins and carcinogens (e.g. carbon monoxide, tar, ammonia and hydrogen cyanide that are typically inhaled when smoking cannabis). However current evidence suggests vaping cannabis poses significant behavioral, psychological, and neurocognitive consequences associated with misuse and addiction [8, 15, 16]. Similarly, cannabis edibles, which are largely perceived as not harmful, possess unique risks such as over-intoxication and increased risks for child-poisoning due to their attractive packaging [5]. There is only modest available evidence on the health benefits that might accrue from use of cannabis in its different forms [8, 15, 16].

Problematic cannabis use (PCU), defined as use that leads to negative health and/or social consequences [17], is a concern among young adults given the changing policy and product landscape. PCU, is a subclinical threshold of use to detect whether cannabis users are experiencing immediate harms and at increasing risk of experiencing future harms due to cannabis use [18, 19]. Several studies have identified frequency of cannabis use, social contexts of use, cannabis use reasons, beliefs, and attitudes as factors associated with problematic cannabis use [20–22]. A study by Hansen et al., found that individuals who self-medicate with cannabis to treat symptoms of inflammatory bowel disease were more likely to experience depression and vulnerability to substance misuse (operationalized as higher scores on the Substance use Risk Profile measure) [23]. To contribute to a comprehensive understanding of how specific health-focused motivations relate to problematic use, and provide scientific evidence to highlight the need for appropriate regulations and policies that strike a balance between providing access to cannabis for health-related reasons while mitigating the potential risks associated with PCU, there is a need for studies that examine broad health-focused reasons for cannabis use and their associations with risk for PCU, especially among vulnerable populations, such as young adults.

The proliferation of unvalidated information on the internet and social media about the potential health benefits of cannabis [24], coupled with increasing accessibility and diversity of cannabis products, is driving an urgent need for practitioners, researchers, and policymakers to understand the effects and risks associated with cannabis use for self-proclaimed health reasons [1, 3]. While some health-focused reasons (e.g., nausea) require infrequent use, some other reasons (e.g., chronic pain) require continued use which could exacerbate or lead to other adverse outcomes including PCU. Hence, research is needed to evaluate the PCU risk associated with cannabis use for these different categories of health-focused reasons most commonly highlighted as motivations for cannabis use. To fill this gap, this study examined the relative risk for PCU associated with different health-focused reasons for cannabis use among young adult cannabis users. We aimed to test the hypothesis that all health-focused reasons for cannabis use is associated with PCU, but participants using for physical health reasons (e.g., chronic pain) will be at highest risk.

## Methods

### Participants and procedures

Data for this study were obtained from a longitudinal survey of substance use behavior and mental health among students from public high schools in Los Angeles County [25]. Approximately 40 schools were invited to participate in the study and 10 schools agreed to participate. All 9th grade students who provided assent and had written parental consent were eligible to be included in the study cohort. Survey administration began during the fall of 9th grade in 2013 where a total of 3,396 students were enrolled in the cohort and surveyed every six months. Following graduation, participants were contacted and those who consented to participate were surveyed annually as young adults (M[SD] age = 21.82[0.38] years). This study used cross-sectional data from young adults who participated in a recent data collection wave (2021) in which cannabis use purposes and PCU were both measured. A total of 2,207 participants provided complete data on past 6-month cannabis products use. Of these participants, 954 reported use of one or more cannabis products and constituted the final analytic sample for the study. All participants provided written informed consent. A university-based Institutional Review Board approved the study.

### Measures

*Cannabis use*. Participants self-reported their past 6-month cannabis use. They were asked if they had “used the following substances in the past 6 months”: blunts (marijuana rolled in tobacco leaf or cigar casing); smoking marijuana (e.g. pot, weed, hash, reefer, bud, grass); electronic device to vape marijuana, THC, or hash oil; marijuana or THC foods or drinks; and dabbing or vaping marijuana concentrates. Only those who answered “yes” to any past 6-month cannabis use were included in the current study.

#### Independent variables

*Cannabis use purposes.* To assess cannabis use purposes among those who indicated any cannabis use in the past 6 months, participants were asked, “*Which of these have you ever used marijuana for?*” Participants were allowed to select one or more of the following purposes: (1) Stress, anxiety, or depression; (2) Chronic pain; (3) Post-traumatic stress; (4) Nausea or low appetite; (5) Insomnia/sleep; (6) Other physical problems (e.g., epilepsy, muscle spasms); (7) Other [with the option to write in a response]; (8) None of the above. From the eight possible response options, three categories of cannabis use purposes were constructed. Physical health purposes included using cannabis to help with ≥ 1 of the following: chronic pain, physical problems, and nausea. Mental health purposes included using cannabis to help with > 1 of the following: stress, anxiety or depression, and post-traumatic stress disorder (PTSD). Sleep health purposes included using cannabis for insomnia and/or sleep issues. The categories for each health theme are non-mutually exclusive binary variables. Participants who did not indicate using cannabis for any health-related reason (e.g., those who indicated only using exclusively for recreation, or “for fun”) were excluded in keeping with study focus on health-related use.

#### Dependent variable

*Cannabis Abuse Screening Test (CAST).* This study used the CAST to assess risk for problematic cannabis use. CAST is a widely applied screening tool for detecting a wider spectrum of problematic use of cannabis in the general population [6, 26]. It has been validated against the Diagnostic and Statistical Manual of Mental Disorders (DSM)-5 and found to screen for patterns that are not detected by the DSM-5 [27]. CAST consists of six items that are answered using a five-point Likert scale (range: 0 to 4). Items assess the frequency of different events in the previous 12-months. The six items are: (1) “*Have you ever used cannabis before noon?*”; (2) “*Have you ever used marijuana when you were alone?*; (3) “*Have you ever had memory problems when you used cannabis?*”; (4) “*Have friends or members of your family ever told you that you should reduce your marijuana use?*”; (5) “*Have you ever tried to reduce or stop your marijuana use without succeeding?*”; (6) “*Have you ever had problems because of your use of marijuana (argument, fight, accident, bad result at work or school, etc.)?*”. Responses to the six CAST items are summed, and the total score (maximum of 24) is used to classify individuals into different risk thresholds. Given the lack of consensus in the literature on the threshold that is most sensitive to identifying problematic cannabis use among young adults,[28] two different CAST levels were examined. The first, a three-threshold structure, referred to as *CAST-3*, used cutoff points suggested by Legleye and colleagues [27], which recommended cut points of ≤ 4 for low risk, 5–7 for moderate risk, and ≥8 for severe risk. The second, a two-threshold structure, referred to as *CAST-2*, used cut off points suggested by Cuenca-Royo and colleagues,[29] which recommended cut points of ≥7 for moderate/severe risk. Both CAST structures have demonstrated sound psychometric properties and reliability in previous studies [6, 27–29]. Specifically, in this study, the Cronbach’s alpha value for the CAST scale was 0.83, indicative of strong reliability. Due to similarity in results, we report results from *CAST-3* and present *CAST-2* results in a supplement table.

### Health problems covariates

Given that mental health and physical comorbidity are associated with cannabis use disorder, we controlled for the effect of present health problems (mental, physical, sleep). Participants indicated (yes or no) if they were prescribed medications for pain relief, emotional or psychological condition, or sleeping aid. Responses to these questions were combined into one binary variable where 0 = no use and 1 = presence of ≥ 1 mental/physical/sleep problems.

### Sociodemographic covariates

Sociodemographic covariates that were included in all analyses were: age (in years), gender (male, female), race/ethnicity (Hispanic/Latino, Non-Hispanic Asian, Non-Hispanic Black, Non-Hispanic White, Non-Hispanic Other), and socioeconomic context (lives comfortably, meets needs with a little left, just meets basic expenses, does not meet basic expenses). A description of all variables in the study is presented in Table 1.

**Table 1:** Descriptive characteristics of study sample

	N (%)
Total participants	954
Sex	
Female	610 (63.94)
Male	337 (35.32)
Missing	7 (0.73)
Race/ethnicity	
Hispanic	524 (54.93)
Non-Hispanic	
White	115 (12.05)
Asian	129 (13.52)
Black	34 (3.56)
Another	72 (7.55)
Missing	80 (8.39)
Socioeconomic context	
Don’t meet basic expenses	59 (6.18)
Just meet basic expenses	237 (24.84)
Meets needs with a little bit left	301 (31.55)
Live comfortably	331 (34.70)
Missing	26 (2.73)
Health problems	
Yes	174 (18.24)
No	753 (78.93)
Missing	27 (2.83)
Cannabis use purposes*	
Physical health	370 (38.78)
Mental health	700 (73.38)
Sleep health	591 (61.95)
# of Cannabis use purposed endorsed	
None	173 (18.13)
One	202 (21.17)
Two	278 (29.14)
Three	301 (31.55)
CAST Mean (SD)	6.33 (5.09)
CAST-3	
Low risk (0–4)	409 (42.87)
Moderate risk (5–7)	199 (20.86)
Severe risk (≥ 8)	346 (36.27)

Note: “Another” race/ethnicity includes: American Indian/Alaska native, Native Hawaiian/Pacific Islander, multiracial, other

* Categories are not mutually exclusive, percentages do not add up to 100

### Statistical methods

The main research question was to examine the association of cannabis use purposes with relative risk for problematic cannabis use among young adult cannabis users. Multinomial logistic regression was used to test associations of cannabis use purpose with CAST-3. Given the lack of consensus on the threshold most suitable to identify problematic cannabis use (i.e., CAST-2 vs. CAST-3), we also examined associations with CAST-2 (see supplement table) in order to ascertain sensitivity of both measurement levels for our study population. All models adjusted for the presence of mental/physical/sleep problems, sociodemographic covariates of age, gender (male as referent), race/ethnicity (non-Hispanic White as referent), and socioeconomic context (live comfortably as referent). Table 2 reports the adjusted relative risk ratios and their corresponding 95% confidence intervals (95% CIs). Adjusted relative risk ratios are considered significant when p < 0.05. All analyses and variables processing were performed with Stata version 17.

**Table 2:** Bivariate tests of associations between CAST score levels and cannabis use purposes

	Physical health			Mental health			Sleep health		
CAST score levels	Yes N (%)	No N (%)	Test Statistic	Yes N (%)	No N (%)	Test Statistic	Yes N (%)	No N (%)	Test Statistic
Low risk	77 (20.81)	332 (56.85)	$\chi$^2^(2) = 146.22 p < 0.01	235 (33.57)	174 (68.50)	$\chi$^2^(2) = 96.74 p < 0.01	187 (31.64)	222 (61.16)	$\chi$^2^(2) = 82.42 p < 0.01
Moderate risk	79 (21.35)	120 (20.55)		160 (22.86)	39 (15.35)		141 (23.86)	58 (15.98)	
High risk	214 (57.84)	132 (22.60)		305 (43.57)	41 (16.14)		263 (44.50)	83 (22.87)	

## Results

As shown in Table 1, the majority of participants were female (63.9%) and of Hispanic/Latino origin (54.9%) with less than 1% and 8.39% missing data on sex and race/ethnicity respectively. About one-third of young adults in this study lived comfortably (34.7%) or were able to meet their needs with a little bit left (31.6%) and 2.73% were missing data on their socioeconomic context. Cannabis use for mental health purposes (e.g., anxiety, stress, depression, PTSD) was the most cited reason in the study sample (73.4%) followed by sleep (61.95%) and physical health (38.78%). As shown in Tables 1 and 36.3% of participants were classified as being at severe risk (CAST score ≥ 8), 20.8% were at moderate risk (5–7 CAST score), and 42.87% at low risk ( 0–4 CAST score). A third (31.55%) of cannabis users in the study endorsed using cannabis for all three use purposes (i.e. mental, sleep, and physical health), 29.14% endorsed two use purposes, and 21.17% endorsed only one use purpose. Among study participants, combustible cannabis was the most used cannabis product type (80%) followed by vaping (69.81%), edibles (68.24%), blunts (54.72%), and concentrates (39.31%).

A Chi square test of independence was performed to assess the relationship between cannabis use purposes and PCU risk (Table 2). There was a significant association between PCU risk and reporting cannabis use for physical health (χ^2^[2] = 146.22, p < 0.01), mental health (χ^2^ [2] = 96.74, p < 0.01), and sleep health (χ^2^ [2] = 82.42, p < 0.01).

Table 3 displays results from the multinomial regression analysis using CAST-3 as the outcome variable to examine the association of cannabis use purposes with the risk for problematic cannabis use. Relative to young adults who did not use cannabis for physical health purposes those who did were 1.8 times more likely to report moderate problematic cannabis use (95% CI = 1.13, 2.74) and 4.4 times more likely to report severe problematic cannabis use (95% CI = 2.96, 6.48). Those who used cannabis for mental health purposes were 2.15 times more likely to report moderate problematic cannabis use (95% CI = 1.32, 3.48) and 2.81 more likely to report severe problematic cannabis use (95% CI = 1.86, 4.72) relative to those who did not endorse cannabis use for mental health reasons. Those who used cannabis for sleep health purposes were 1.9 times more likely to report moderate problematic cannabis use (95% CI = 1.22, 3.00) and 1.83 times more likely to report severe problematic cannabis use (95% CI = 1.21, 2.77) reltive to those who did not use cannabis for sleep health reasons. Results obtained using CAST-3 were generalizable to CAST-2 which are presented in supplement Table 1. We also examined the association of each of the eight individual cannabis use reasons and results are presented in supplement Table 2.

**Table 3:** Multivariable regression analysis testing association of cannabis use purpose and risk for problematic cannabis use (CAST-3)

	Moderate PCU risk					Severe PCU risk			
	Coeff.	ARR	95% CI*	P		Coeff.	ARR	95% CI*	P
Physical health	**0.57**	**1.76**	1.13–2.74	0.012		**1.48**	**4.38**	2.96–6.48	< 0.001
Mental health	**0.76**	**2.15**	1.32–3.48	0.002		**1.03**	**2.81**	1.75–4.50	< 0.001
Sleep health	**0.64**	**1.90**	1.22–3.00	0.005		**0.61**	**1.83**	1.21–2.77	0.004
Health problems (yes)	-0.16	0.86	0.50–1.45	0.564		0.45	1.56	0.99–2.45	0.053
Race (ref = White)									
Hispanic	-0.42	0.66	0.37–1.16	0.147		0.24	1.12	0.74–2.19	0.387
Asian	-0.19	0.83	0.41–1.67	0.594		0.11	1.00	0.56–2.19	0.761
Black	0.20	1.23	0.43–3.50	0.645		0.43	1.37	0.55– 4.35	0.413
Other	-0.35	0.71	0.33–1.53	0.418		-0.76	0.47	0.21–1.07	0.072
Age	0.28	1.32	0.78–2.24	0.307		**0.49**	**1.59**	0.99–2.56	0.049
Sex (Female)	**-0.52**	**0.59**	0.40–0.89	0.012		**-0.64**	**0.54**	0.36– 0.77	0.001
SES context (ref = live comfortably)									
Don’t meet basic expenses	**1.16**	**3.19**	1.28–7.98	0.013		**1.31**	**3.72**	1.54–8.96	0.003
Just meet basic expenses	0.37	1.45	0.90–2.37	0.137		**0.62**	**1.86**	1.17–2.96	0.009
Meets needs with a little bit left	-0.27	0.76	0.48–1.21	0.249		0.07	1.08	0.71–1.66	0.732

*95% Confidence Interval for adjusted relative risk ratio

PCU: Problematic cannabis use

Coeff: Multinomial Regression coefficient

ARR: Adjusted relative risk ratio

Base/Referent outcome category: Low risk

Bold values indicate statistical significance, p < 0.05

Regarding sociodemographic covariates (Table 3), for females relative to males, they were 0.6 times less likely to report moderate and severe PCU risk. Reagrding socioeconomic context, specifically, those who did not meet their basic expenses were about 3 times (95% CI = 1.28, 7.98) and 4 times (95% CI = 1.54, 8.96) more likely to report moderate and severe PCU risk relative to those who live comfortably (highest socioeconomic context).

## DISCUSSION

The current study examined the relationship of different health-focused cannabis use purposes with problematic cannabis use (PCU) risk. More than half of study participants who reported past 6-month cannabis use were classified as having moderate to severe risk for problematic cannabis use. Mental health reasons (stress, anxiety, depression, PTSD) was the most endorsed reason for use among study sample. Overall, findings demonstrate an increased risk of PCU among all users irrespective of health-focused use purposes. However, those who endorsed using cannabis due to physical health reasons (including use of cannabis for chronic pain, physical problems such as muscle spasm, and nausea) had the highest relative risk for severe PCU. Those who indicated using cannabis due to sleep reasons had an increased risk for severe PCU but the risk estimates were not as robust as those associated with mental and physical health.

Chronic pain continues to impact children [30] and adults [31] with a recent review reporting 11.6% of young adults experience chronic pain [30]. Effective chronic pain pharmaceutical treatment options have limited effectiveness and well-known safety concerns, and cannabis use for pain management is becoming increasingly common [3] fueling the increased public perception of the effectiveness of cannabis for ameliorating chronic pain. Current evidence shows that inhaled cannabis is effective for pain management among adults [32]. There is also moderate evidence that oromucosal cannabinoids improve short-term sleep disturbances associated with chronic pain. The two main active components in cannabis are delta-9-tetrahydrocannabinol (THC) and cannabidiol (CBD) which interact to produce the analgesic and psychotropic effects of cannabis depending on the pharmacokinetic formulation and route of administration. Although scientific advancements have allowed for extraction of pure cannabis extracts on order to develop drugs specifically tailored to leverage the analgesic effect of the cannabis plant (i.e., from THC), the largely unregulated cannabis product landscape makes it difficult for users who self-medicate with cannabis to identify what product formulation is most suitable for their ailment and will not increase their risk for problematic use. The expanding yet variable legalization, lack of regulation and consistency in cannabis product formulation (e.g., active ingredient concentrations), diverse landscape of cannabis products and routes of administration, and the modest impact of cannabis for long term pain management could be responsible for the increased relative risk of severe PCU among users of cannabis products for physical problems.

Cannabis products designed for use in medicinal and recreational purposes are very different, yet an overlap now exists owing to limited scientific evidence and product proliferation. In randomized control trials of medicinal use of cannabis for chronic pain, reported THC levels ranged from 0 − 9.4% and almost all the studies reported adverse side effects from study participants even at those levels [32]. In contrast, recreational formulations could have THC concentrations as high as 30%, and such formulations are also now marketed as having medicinal potencies for treating various ailments. For example, well-known websites (e.g., www.leafly.com) market myriad cannabis varieties and preparations some of which have up to 22% THC and are also marketed to medical marijuana patients as useful for relieving pain, stress and depression. This significant overlap in marketing, promotion, and use of recreational cannabis products for medicinal purposes could explain why all the different use purposes examined in the study were associated with increased PCU risk.

It is also possible that increased PCU risk associated with all reported health reasons for cannabis use could be attributed to use of cannabis for negative reinforcement, i.e. cannabis is used to reduce negative affect from health-related stressors such physical pain, mental health problems, and sleep issues. Previous studies have found that individuals with higher negative affect used cannabis for coping (using cannabis to regulate negative emotions) in order to increase positive affect (feelings of relief, joy, happiness) [33, 34]. It is possible individuals who use cannabis and then get some sort or acute symptom amelioration will get reinforced. However, the cannabis may not have any lasting therapeutic effect because, unlike an anti-depressant, it is a short-acting psychotropic drug. This could promote a cycle of use to constantly suppress unpleasant symptoms thereby increasing PCU risk.

Despite the important contribution that this study makes, findings should be considered in the context of their strengths and limitations. While the study strengths include the use of a relatively large and racially/ethnically diverse sample of young adult cannabis users, participants were predominantly Hispanic/Latino from a large metropolitan area, hence, the results may not be generalizable to all young adults nationally. Another study strength is examination of two different PCU risk thresholds (CAST-2 and CAST-3) to ensure sensitivity. Despite this, it is important to note that one of the CAST questions asks if participants “*have ever used cannabis before noon*”. Participants who use cannabis for insomnia may report lower frequency which could result in the measure being less sensitive to PCU risk among those who frequently use later in the day. Another limitation is that the study employed self-reported measures which are subject to social desirability and recall bias. Additionally, the cannabis use purposes overlapped as participants were able to select more than one use purpose (only 21% of participants endorsed just one cannabis use purpose). Furthermore, the data were examined cross-sectionally, thereby precluding the examination of longitudinal patterns of different cannabis use motivations on PCU risk. As a result, findings caanot be be interepreted as causal. Also, although data missingness was low, the use of listwise deletion, albeit a recommended approach for our study context [35], may have underpowered the study’s ability to detect associations.

## Conclusion

Our study adds to the literature on cannabis use purposes and problematic cannabis use risk by highlighting a number of important considerations for researchers and practitioners. Given the current legalization efforts and cultural acceptance surrounding cannabis use, problematic cannabis use is a concern that would likely increase as the prevalence of users increase [36]. Further, the lack of regulation and clear guidelines on cannabis products designated for medicinal purposes will pose a significant health risk given that those who use cannabis for health-related reasons often do so to alleviate chronic symptoms and are more likely to use daily/frequently [37]. Frequent use of cannabis, popularity of diverse cannabis plant products with varying THC and cannabidiol composition, coupled with smoking/vaping as the most common method of administration could elicit adverse health outcomes especially because cannabis smoke contains bronchial irritants similar to tobacco and is linked with adverse respiratory effects [37, 38].

This study identifies health-related reasons that young adults identify as motives for cannabis use. Furthermore, this study provides insight into the associations of these health-focused cannabis use reasons and problematic cannabis use risk among young adults. These findings provide scientific evidence to demonstrate the risk for cannabis use disorder associated with self-medicating with cannabis, especially in the current lansdacape of cannabis products and policies. More research on routes of administration, cannabis product type, and whether cannabis used was prescribed is needed to further inform policies and regulation surrounding cannabis product design, marketing, and use.

### Electronic supplementary material

Below is the link to the electronic supplementary material.

Supplementary Material 1

## Data Availability

The datasets used and/or analyzed during the current study are available from the corresponding author on reasonable request.
