# Supplementary material for: Association of self-reported use of cannabis for the purpose of improving physical, mental, and sleep health with problematic cannabis use risk
Source: BMC Public Health. 2023 Aug 16;23:1560. doi: 10.1186/s12889-023-16324-0 (PMC10429075; doi:10.1186/s12889-023-16324-0)
Supplement: Supplementary file 1 — Supplementary Material 1 [file 12889_2023_16324_MOESM1_ESM.docx]

Supplement Table 1. Logistic regression analysis testing association of cannabis use purpose and risk for problematic cannabis use (CAST-2)

|  | Severe PCU risk | | |
| --- | --- | --- | --- |
|  | AOR | 95% CI | p |
| Physical health | **3.14** | 2.26 – 4.39 | <0.001 |
| Mental health | **2.40** | 1.57 – 3.65 | <0.001 |
| Sleep health | **1.60** | 1.11 – 2.31 | 0.012 |
| Health problems (yes) | **1.49** | 1.00 – 2.22 | 0.048 |
| Race (ref=White) |  |  |  |
| Hispanic | 1.55 | 0.97 – 2.48 | 0.066 |
| Asian | 1.17 | 0.65 – 2.11 | 0.881 |
| Black | 1.50 | 0.62 – 3.67 | 0.523 |
| Other | 0.56 | 0.28 – 1.14 | 0.077 |
| Age | 1.27 | 0.88 – 2.05 | 0.170 |
| Sex (Female) | 0.80 | 0.58 – 1.11 | 0.183 |
| SES context (ref=live comfortably) |  |  |  |
| Don’t meet basic expenses | **2.71** | 1.32 – 5.56 | 0.007 |
| Just meet basic expenses | 1.45 | 0.97 – 2.16 | 0.068 |
| Meets needs with a little bit left | 1.06 | 0.73 – 1.55 | 0.744 |

PCU: Problematic cannabis use

AOR: Adjusted odds ratio

Base/Referent outcome category: Moderate risk

Bold values indicate statistically significance, p<0.05

Supplement Table 2. Multivariable regression analysis testing association of each of the eight cannabis use purposes and risk for problematic cannabis use (CAST-3)

|  | Moderate PCU risk | | | |  | Severe PCU risk | | | |
| --- | --- | --- | --- | --- | --- | --- | --- | --- | --- |
|  | Coeff. | ARR | 95% CI* | P |  | Coeff. | ARR | 95% CI* | P |
| Stress/Anxiety/Depression | 0.41 | 1.51 | -0.15 – 0.97 | 0.151 |  | **0.85** | **2.35** | 1.32 – 4.19 | 0.004 |
| Chronic pain | 0.01 | 1.01 | -0.54 – 0.56 | 0.971 |  | 0.22 | 1.25 | 0.76 – 2.03 | 0.380 |
| Post-traumatic stress | 0.27 | 1.31 | -0.52 – 1.06 | 0.503 |  | 0.36 | 1.44 | 0.72 – 2.89 | 0.305 |
| Nausea/Low appetite | **1.00** | **2.72** | 0.47 – 1.53 | p<0.001 |  | **1.74** | **5.70** | 3.56 – 9.14 | p<0.001 |
| Insomnia/Sleep | **0.51** | **1.67** | 0.04 – 0.99 | 0.035 |  | **0.50** | **1.65** | 1.05 – 2.58 | 0.029 |
| Other physical problems (muscle spasm, epilepsy) | -0.63 | 0.53 | -1.65 – 0.39 | 0.223 |  | 0.19 | 1.21 | 0.55 – 2.67 | 0.630 |
| Other | 0.23 | 1.26 | -0.40 – 0.85 | 0.477 |  | -0.11 | 0.90 | 0.48 – 1.69 | 0.741 |
| None of the above | -0.76 | 0.47 | -1.65 – 0.12 | 0.091 |  | -0.25 | 0.78 | 0.34 – 1.79 | 0.551 |

*95% Confidence Interval for adjusted relative risk ratio

Model adjusts for presence of health problems and sociodemographic covariates

PCU: Problematic cannabis use

Coeff: Multinomial Regression coefficient

ARR: Adjusted relative risk ratio

Base/Referent outcome category: Low risk

Bold values indicate statistical significance, p<0.05.
